# A clinical text classification paradigm using weak supervision and deep representation

**DOI:** 10.1186/s12911-018-0723-6

**Published:** 2019-01-07

**Authors:** Yanshan Wang, Sunghwan Sohn, Sijia Liu, Feichen Shen, Liwei Wang, Elizabeth J. Atkinson, Shreyasee Amin, Hongfang Liu

**Affiliations:** 1 Division of Biomedical Statistics and Informatics, Department of Health Sciences Research, Mayo Clinic, 200 1st ST SW, Rochester, MN 55905 USA; 2 Division of Rheumatology, Department of Medicine, Mayo Clinic, 200 1st ST SW, Rochester, MN 55905 USA; 3 Division of Epidemiology, Department of Health Sciences Research, Mayo Clinic, 200 1st ST SW, Rochester, MN 55905 USA

**Keywords:** Clinical text classification, Natural language processing, Electronic health records, Machine learning, Weak supervision

## Abstract

**Background:**

Automatic clinical text classification is a natural language processing (NLP) technology that unlocks information embedded in clinical narratives. Machine learning approaches have been shown to be effective for clinical text classification tasks. However, a successful machine learning model usually requires extensive human efforts to create labeled training data and conduct feature engineering. In this study, we propose a clinical text classification paradigm using weak supervision and deep representation to reduce these human efforts.

**Methods:**

We develop a rule-based NLP algorithm to automatically generate labels for the training data, and then use the pre-trained word embeddings as deep representation features for training machine learning models. Since machine learning is trained on labels generated by the automatic NLP algorithm, this training process is called weak supervision. We evaluat the paradigm effectiveness on two institutional case studies at Mayo Clinic: smoking status classification and proximal femur (hip) fracture classification, and one case study using a public dataset: the i2b2 2006 smoking status classification shared task. We test four widely used machine learning models, namely, Support Vector Machine (SVM), Random Forest (RF), Multilayer Perceptron Neural Networks (MLPNN), and Convolutional Neural Networks (CNN), using this paradigm. Precision, recall, and F1 score are used as metrics to evaluate performance.

**Results:**

CNN achieves the best performance in both institutional tasks (F1 score: 0.92 for Mayo Clinic smoking status classification and 0.97 for fracture classification). We show that word embeddings significantly outperform tf-idf and topic modeling features in the paradigm, and that CNN captures additional patterns from the weak supervision compared to the rule-based NLP algorithms. We also observe two drawbacks of the proposed paradigm that CNN is more sensitive to the size of training data, and that the proposed paradigm might not be effective for complex multiclass classification tasks.

**Conclusion:**

The proposed clinical text classification paradigm could reduce human efforts of labeled training data creation and feature engineering for applying machine learning to clinical text classification by leveraging weak supervision and deep representation. The experimental experiments have validated the effectiveness of paradigm by two institutional and one shared clinical text classification tasks.

## Background

The initiation of the Health Information Technology for Economic and Clinical Health Act (HITECH Act) in 2009 has fostered the rapid adoption of Electronic Health Record (EHR) systems at US hospitals and clinics. The number of healthcare organizations with a fully operational EHR system has increased to 22% in 2010, compared to 17% in 2009 [1]. Large amounts of detailed longitudinal patient information, including lab tests, medications, disease status, and treatment outcomes, has been accumulated electronically and becomes valuable data sources for clinical and translational research [2–4]. A well-known challenge faced when using EHR data for research is that large amounts of detailed patient information is embedded in clinical text (e.g., clinical notes and progress reports). Automated clinical text classification, one of the popular natural language processing (NLP) technologies, can unlock information embedded in clinical text by extracting structured information (e.g. cancer stage information [5–7], disease characteristics [8–10] and pathological conditions [11]) from the narratives. Many successful clinical studies applying clinical text classification have been reported, including phenotyping algorithms [12, 13], detection of adverse events [14], improvement of healthcare quality [15, 16] and facilitation of genomics research [17–20].

Clinical text classification tasks can be tackled using either symbolic techniques or statistical machine learning [21]. Applications built based on symbolic techniques involve handcrafted expert rules, such as regular expressions and logic rules, implemented in rule-based NLP tools, such as MedTagger [22]. It has been shown effective in the clinical domain due to the clinical sublanguage characteristics [23]. However, rule-based applications can be labor expensive and cumbersome to develop, requiring collaboration between NLP experts and healthcare professionals, and the resultant applications may not be portable beyond the use case for which it is designed.

Machine learning approaches have been shown to be efficient and effective for clinical text classification tasks [24, 25]. Despite the impressive improvement in these tasks, a successful machine learning model usually requires extensive human efforts to label a large set of training data. This problem becomes more significant in the clinical domain, mainly due to i) the lack of publicly available clinical corpora due to privacy concerns, and ii) the requirement of medical knowledge to accurately annotate clinical text. Therefore, popular methods for creating labeled training data, such as crowdsourcing, are not applicable for clinical information extraction tasks.

In the literature, researchers have utilized the weak supervision strategy to train machine learning models on the weakly labeled training data created by automated methods. Weak supervision is a simple and adaptable approach leveraging programmatically created weakly labeled training sets. It is proposed primarily for relation extraction from text, wherein a known relation from a knowledge base (e.g. Freebase) is likely to express that relation in an input corpus [26, 27]. Furthermore, weak supervision has been widely applied in other common NLP tasks including knowledge-base completion [28], sentiment analysis [29], and information retrieval [30]. In the biomedical domain, weak supervision has been used to augment machine learning based classifiers to identify drug-drug interactions or medical terms from biomedical literature [31–34]. In the clinical domain, Wallace et al. [35] proposed a weak supervision approach to better exploit a weakly labeled corpus to extract sentences of population/problem, intervention, comparator, and outcome from clinical trial reports.

In addition to labeled training data creation, feature engineering, which is fundamental to machine learning, also requires considerable human efforts. In order to enable machine learning methods to process raw text data, we need careful feature engineering to transfer the raw data into feature vectors. Recently, the deep representation learning has become popular due to its capability to represent raw data as a high level feature vector and due to its independence from the classification task [36]. In NLP, word embeddings are one of the most successful deep learning technologies with the ability to capture high-level semantic and syntactic properties of words [37–39]. Word embeddings have been utilized in various clinical NLP applications, such as clinical abbreviation disambiguation [40], named entity recognition [41], and information retrieval [42]. Henriksson et al. [43] leverage word embeddings to identify adverse drug events from clinical notes. Their experiments show that employing word embeddings could improve the predictive performance of machine learning methods. Both Tang et al. [44] and Wu et al.’s [41] studies show that word embedding features outperform other features for clinical named entity recognition. In addition, word embeddings help improve the relation extraction, such as relations between medical problems and treatments, relations between medical problems and tests, and relations between medical problems and medical problems, in clinical notes [45]. However, to the best of our knowledge, there are no clinical applications in the literature utilizing word embeddings as features for weak supervision. In particular, no study utilizes rule-based NLP for generating weakly labeled training data for machine learning methods and uses word embeddings as features. Our hypothesis is that deep representation using word embeddings might enable machine learning methods to learn extra patterns from weakly labeled training data and outperform rule-based NLP systems used to generate the weak labels since it could then find semantically similar words in embedding space while these words may not be included in the NLP rules.

In this study, we propose a clinical text classification paradigm using weak supervision and deep representation to reduce human efforts for the labeled data creation and feature engineering. The proposed paradigm utilizes the rule-based NLP algorithms to automatically generate weak labels for training data. We then use the pre-trained word embeddings as deep representation features to eliminate the need of task-specific feature engineering for training machine learning models. Since the machine learning models are trained on labels generated by the NLP algorithm instead of human annotators, this training process is called weak supervision. To illustrate the effectiveness of the proposed paradigm, we conducted empirical experiments on two institutional case studies at Mayo Clinic: smoking status classification and proximal femur (hip) fracture classification, and one case study using a public dataset: the i2b2 2006 smoking status classification shared task. We tested four widely used machine learning models in the paradigm, namely, Support Vector Machine (SVM), Random Forrest (RF), Multilayer Perceptron Neural Networks (MLPNN), and Convolutional Neural Networks (CNN), and the advantage of word embedding features in the proposed paradigm. Furthermore, we showed the impact of the training data size on the performance of machine learning methods.

## Methods

**Fig. 1:**
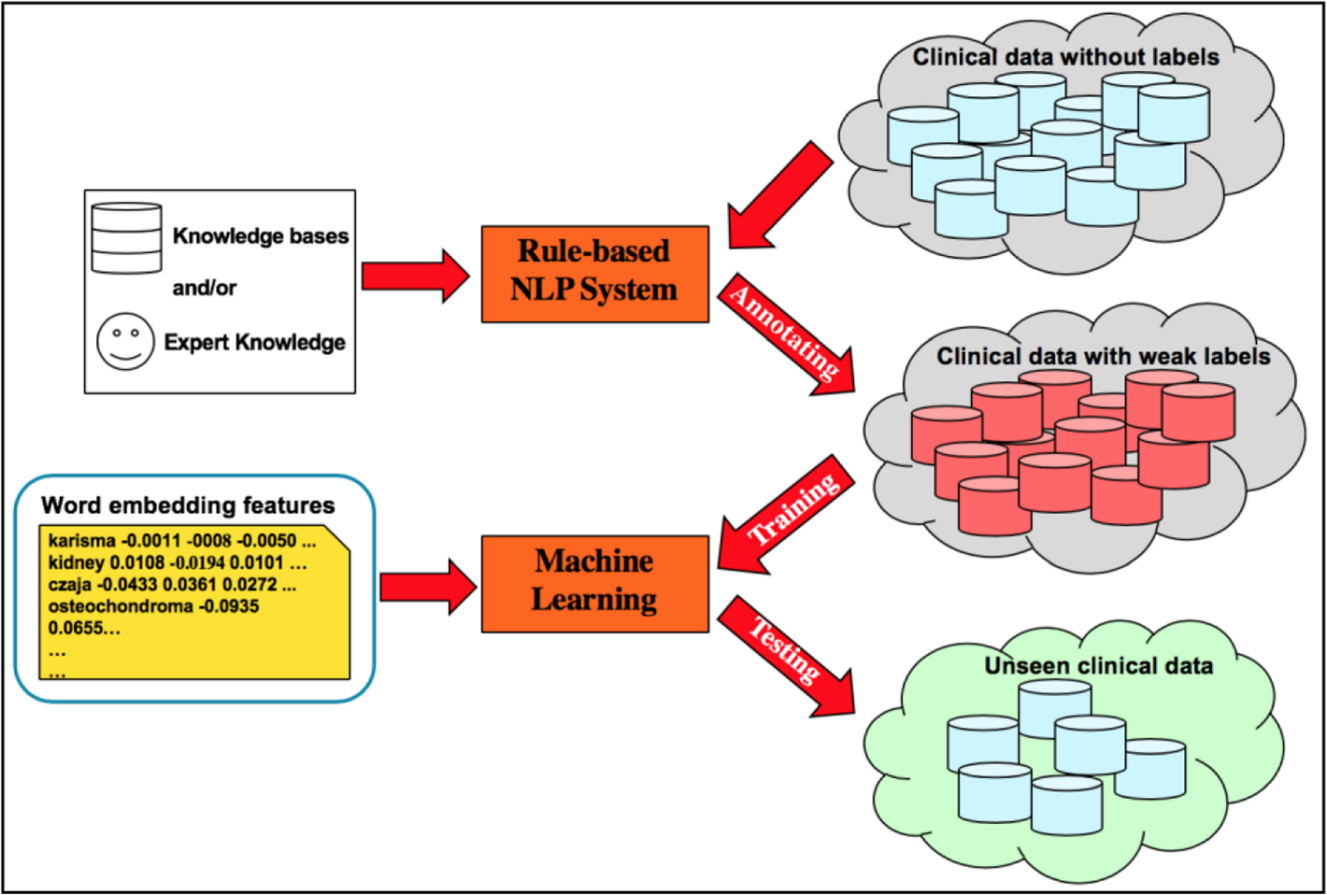
The schema of clinical text classification paradigm using weak supervision and deep representation. Note: The clipart in this figure is designed by the authors

We here describe the proposed clinical text classification paradigm using weak supervision and deep representation. Figure 1 illustrates the schema of the proposed paradigm. In the first step, a rule-based NLP algorithm is developed based on expert knowledge and experience, and then applied on non-labeled clinical text to automatically generate weak labels. By doing so, one can create a large set of weakly labeled training data quickly. In the second step, we use the pre-trained word embeddings to map each instance into a deep semantic vector representation, and adopt weak supervision to train machine learning methods using the deep representations as input and the corresponding weak labels as learning objectives. Eventually we utilize the trained machine learning model to extract information from unseen clinical text.

In the following, we theoretically prove that training machine learning models using weak supervision approximates training with the true labels in terms of performance. We define the rule-based NLP algorithm as *λ* containing *m* rules, i.e., *λ*_*i*_ : *d* ⟼ {*y*_1_, *y*_2_, …, *y*_*L*_}, where *λ*_*i*_, *i* = 1, 2, …, *m* is the *i*th rule and *d* is the clinical document, {*y*_1_, *y*_2_, …, *y*_*L*_} is a set of labels for the document *d*. Here, for simplicity, we assume that the task is a binary classification (i.e., *λ*_*i*_ : *d* ⟼ {1, −1}). Suppose that each rule is a function *λ*_*i*_ that has the probability *ϕ*_*i*_ of labeling the document correctly, we can write the distribution of the rule-based NLP algorithm as below:

1 $$ {p}_{\phi}\left(\Lambda, Y\right)=\frac{1}{2}\prod \limits_i^m\left({\phi}_i{1}_{\left\{\varLambda =Y\right\}}+\left(1-{\phi}_i\right){1}_{\left\{\varLambda \ne Y\right\}}\right), $$

where *Λ* is the label output by the NLP algorithm, *Y* is the true label, and each label is assumed to be uniformly distributed.

Suppose that the word embedding features are generated from a mapping function *f*, we can write the empirical loss function *L*_*ϕ*_(*w*) of using the weak labels as:

2 $$ {L}_{\phi }(w)=\frac{1}{\left|D\right|}\sum \limits_{d\in D}{E}_{\left(\Lambda, Y\right)\sim {p}_{\phi }}\left[\log \left(1+\exp \left(-{w}^Tf(d)Y\right)\right)|\varLambda =\lambda (d)\right]+\rho {\left\Vert w\right\Vert}^2, $$

where *D* is the clinical document set, |*D*| is the number of documents in *D*, *w* is a parameter and *ρ* is a *l*_2_ regularization parameter. Similarly, we can write the loss function of using true labels as:

$$ L(w)=\frac{1}{\left|D\right|}\sum \limits_{d\in D}{E}_{\left(\Lambda, Y\right)\sim p}\left[\log \left(1+\exp \left(-{w}^Tf(d)Y\right)\right)\right]+\rho {\left\Vert w\right\Vert}^2, $$

where *p* is the distribution of true labels. We assume that there are *m* = *Ο*(1) rules and |*D*| = *Ο*(*ϵ*^−2^) training data where *ϵ* is the parameter estimate error. According to the mean value theorem and Cauchy-Schwarz inequality [46], we can derive an upper bound for the difference between the loss function using true labels (i.e., *L*(*w*)) and that using weak labels (i.e., *L*_*ϕ*_(*w*)), i.e.,

$$ \left|L(w)-{L}_{\phi }(w)\right|\le \frac{c\left\Vert w\right\Vert \epsilon }{2} $$

where *c* is a constant value. Since *w* generally satisfies $\left\Vert w\right\Vert \le \frac{1}{2\rho }$, the upper bound is small enough. Thus, the weak supervision addresses the problem of lacking large labeled training data for machine learning models without hurting the performance of clinical text classification.

**Fig. 2:**
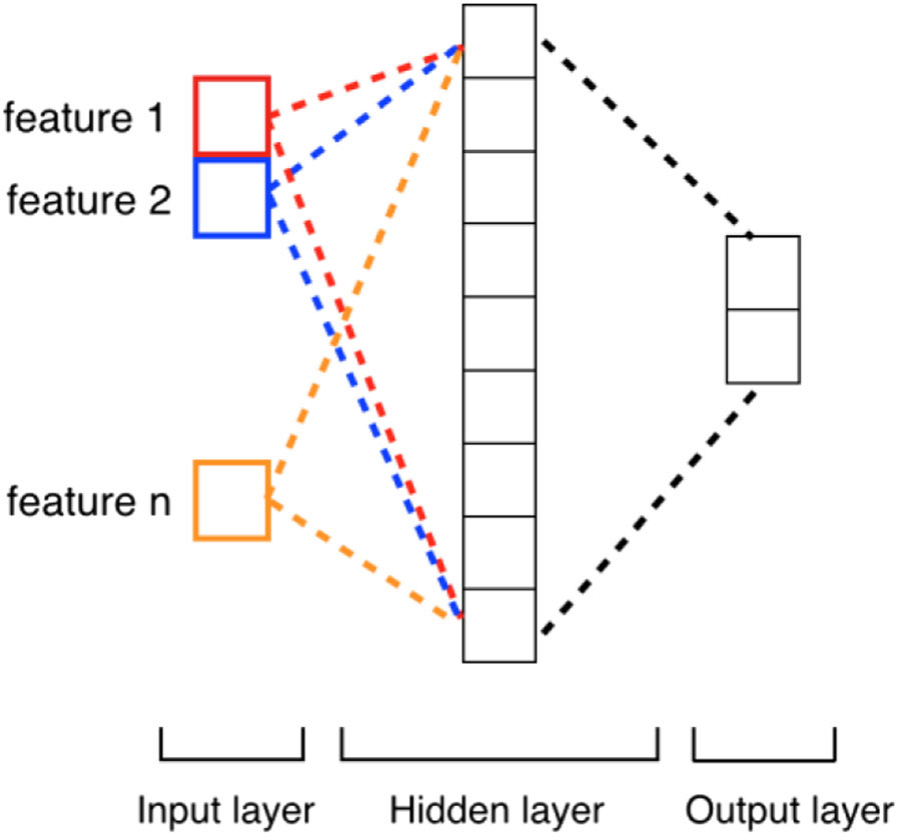
Architecture of the MLPNN model

**Fig. 3:**
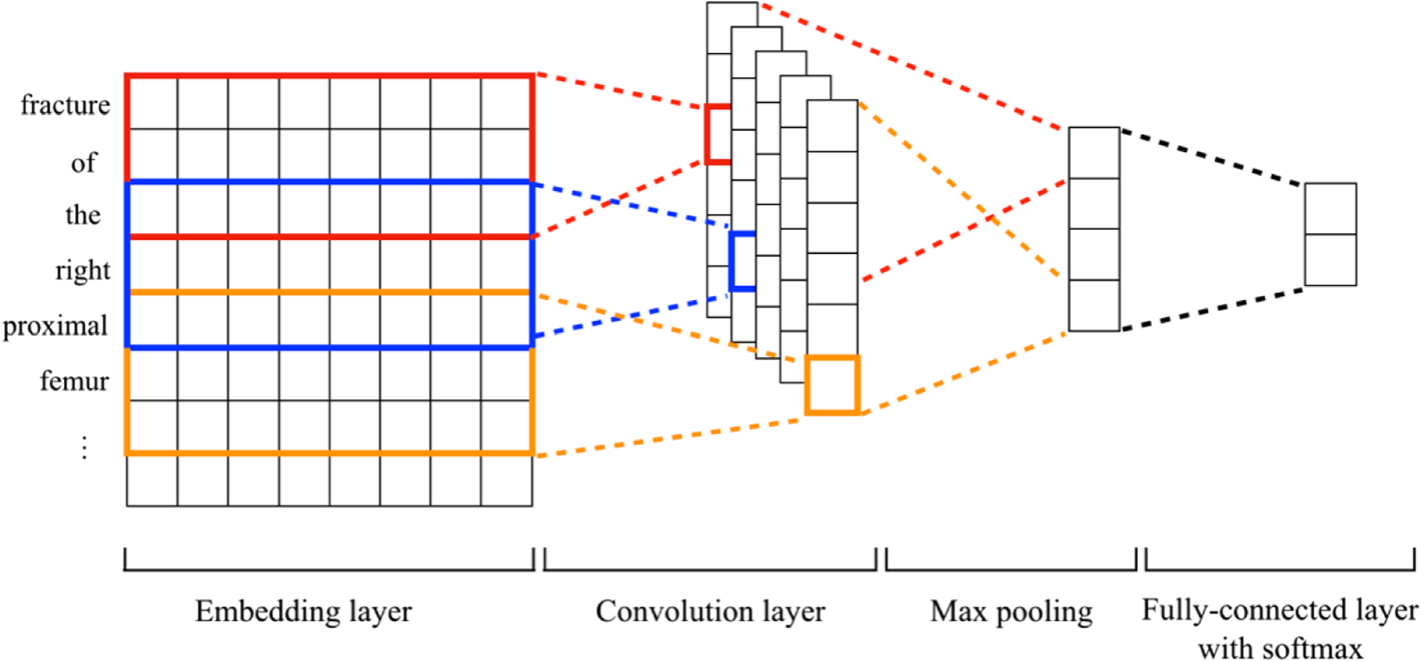
Architecture of the CNN model

Although any machine learning method can be applied in the proposed paradigm, we would like to investigate which model fits better in the paradigm. In this study, we tested four prevalent machine learning methods, namely Support Vector Machine (SVM), Random Forrest (RF), Multilayer Perceptron Neural Networks (MLPNN), and Convolutional Neural Networks (CNN). SVM is a supervised learning method that has been widely used for classification [47]. We utilized linear SVM and set the parameter C to 10 in our experiments. RF is an ensemble of classification trees, where each tree contributes with a single vote for the assignment of the most frequent class to the input data [48]. Compared to SVM, RF has high classification accuracy and ability to model complex interactions among input variables. In our experiment, we set the number of trees in the forest to 5 for RF. MLPNN is a class of feed-forward artificial neural networks consisting of at least three layers of nodes: an input layer, a hidden layer and an output layer. The architecture of MLPNN is shown in Fig. 2. As a comparison to deep neural networks, we applied a single-layered MLPNN, set the number of neurons to 15 and used the rectified linear unit function as the activation in our experiments. CNN is a specific architecture of MLPNN with deep hidden layers formed by a convolution operation followed by a pooling operation [49]. In our experiment, we used a CNN model consisting of embedding layer, convolution layer and fully-connected layer with a softmax function, as shown in Fig. 3. We tested filter sizes of 128, 256, 512, and chose 128 as the filter size since it had the best performance. We utilized the categorical cross entropy as loss function, the rectified linear unit (ReLU) as activation function, and the root mean square propagation (RMSprop) as gradient descent optimization algorithm in the CNN.

In addition to the large set of automatically generated labeled training data using the proposed paradigm, we used word embeddings as a deep representation of words to capture high-level semantic and syntactic properties. The word embeddings used in our experiments were trained using word2vec [50] on a large corpus consisting of the textual clinical notes for a cohort of 113 k patients receiving their primary care at Mayo Clinic, spanning a period of 15 years from 1998 to 2013 [51]. We set the dimension of word embeddings to 100 since our previous study shows that word embeddings with the dimension size of 100 can best represent medical word semantics [51]. For CNN, the pre-trained word embeddings are directly utilized to map words into vectors in the embedding layers. In order to obtain the feature of each instance for SVM and RF, we calculated the mean of the summation of word embeddings of words in the instance. Specifically, given an instance *d* = {*w*_1_, *w*_2_, .., *w*_*M*_} where *w*_*i*_, *i* = 1, 2, …, *M* is the *i*th word and *M* is the total number of words in this instance, the feature vector **x** of instance *d* is defined by:

3 $$ \mathbf{x}=\frac{1}{M}\sum \limits_i^M{\mathbf{x}}_i, $$

where **x**_*i*_ is the embedding vector for word *w*_*i*_ from the word embeddings.

To verify the strength of word embeddings in the proposed paradigm, we compared the performance of word embeddings in SVM and RF with two other popular feature representations: term frequency-inverse document frequency (tf-idf) and topic modeling. Since the layer of word embeddings is a component of the CNN model, this comparison is not conducted on CNN. The tf-idf document representation is a common term weighting scheme in information retrieval, which has been also found effective for document classification [52–54]. It represents a document using a vector with dimension as the vocabulary size of the corpus and elements corresponding to the tf-idf weight of each word *w* in the document *d*. Topic modeling is a widely used semantic representation of document features. To derive document representation using topic modeling, we employed Latent Dirichlet Allocation (LDA) [55], and set the number of topics as 100 to be consistent with the dimension of word embeddings. The document representation was then derived similarly to word embeddings where *x*_*i*_ in Eq. (3) becomes the word-topic mixture distribution of word *w*_*i*_.

Precision, recall, and F1 score were used as metrics to evaluate the performance of the proposed paradigm. All statistical analysis was conducted using t-test at significance level of 5% (*p* = 0.05).

### Materials

We evaluated the effectiveness of the proposed paradigm on two practical binary clinical text classification case studies at Mayo Clinic: smoking status classification and proximal femur (hip) fracture classification, and one case study using a public dataset: the i2b2 2006 smoking status classification shared task. This study was a retrospective study of existing records. The study and a waiver of informed consent were approved by Mayo Clinic Institutional Review Board in accordance with 45 CFR 46.116 (Approval #17–003030).

#### Case study 1: Mayo Clinic smoking status classification

We first examined the proposed paradigm on a smoking status classification task at Mayo Clinic with the aim of identifying the smoking status in a clinical note, i.e., *smoker* (including current smoker and past smoker) or *non-smoker*. We curated a corpus of 32,336 instances by using the “social and behavior history” section from the clinical notes in the Mayo Clinic EHR system [56, 57]. To evaluate the performance, we randomly sampled 475 of them to create a test dataset with the gold standard labels manually annotated by an expert with medical background. For the remaining 31,861 clinical notes, we developed a simple rule-based NLP algorithm, as shown in Table 1, to extract smoking status instances. Note that the smoking status was non-smoker if no information was extracted from a clinical note. By doing so, we created a large weakly labeled training dataset for machine learning models.

**Table 1:** Keywords of the NLP algorithm for the extraction of smoking status

Smoker	smokes?, smoked, smoking, smokers?, tobaccos?, cigarettes?, cigs?, pipes?, nicotine, cigars?, tob
Non-Smoker	(no|non|not|never|negative)\W*(smoker|smoking|smoked|tobacco), nonsmoker, denies\W*smoking, (tobacco|smoke|smoking|nicotine)\W*(never|no), doesn\'t smoke, 0|zero smokers?

#### Case study 2: Proximal femur (hip) fracture classification

In the second experiment, we evaluated the paradigm on a proximal femur (hip) fracture classification task at Mayo Clinic. Among fractures, proximal femur (hip) fractures are of particular clinical interest as they are most often related to bone fragility from osteoporosis, and are associated with significant mortality and morbidity in addition to high health care costs [58]. In this task, a set of 22,969 radiology reports (including general radiography reports, computed tomography reports, magnetic resonance imaging reports, nuclear medicine radiology reports, mammography reports, ultrasonography reports, and neuroradiology reports, amongst others) from 6,033 Mayo Clinic patients were used to determine whether a proximal femur (hip) fracture could be identified using radiology reports [59, 60]. The subjects were aged 18 years of age or older, residents of Olmsted County, and had experienced at least one fracture at some site during 2009–2011. Similar to the previous experiment, we randomly sampled 498 radiology reports as testing data and asked a medical expert with multiple years of experience abstracting fractures to assign a gold standard to each radiology report.

Table 2 shows the rule-based NLP algorithm for this proximal femur (hip) fracture classification task. The rules were developed and refined through verification with physicians and supplemented with historical rules developed by the Osteoporosis Research Program at Mayo Clinic to aid the nurse abstractors in proximal femur (hip) fracture extraction. In this NLP algorithm, the fracture modifiers must appear in the context of keywords within a sentence. We ran this NLP algorithm on the training dataset and obtained a weak label for each document, which was subsequently used to train machine learning models. Finally, we tested the performance on the testing dataset using the gold standard annotated by the medical expert.

**Table 2:** Keywords of the NLP algorithm for the extraction of proximal femur (hip) fracture

Keywords	cervical|femoral head|neck, (trans)?cervical, (sub)?capital, intracapsular, trans(|-)?epiphyseal, base of neck, basilar femoral neck, cervicotrochanteric, (greater|lesser) trochanter, (inter|per|intra) trochanteric
Fracture Modifiers	(micro-?)?fracture(s|d)?, (epi|meta)physis, separation, fxs?, broken, cracked, displace(d)?, fragment

#### Case study 3: i2b2 2006 smoking status classification

In the third case study, we tested the proposed approach on i2b2 2006 smoking status classification shared task with the aim of automatically determining the pre-defined smoking status of patients from information found in their discharge records [61]. These five pre-defined smoking status categories are: *past smoker*, *current smoker*, *smoker*, *non-smoker*, and *unknown*, where a past and current smoker are distinguished based on temporal expressions in the patient’s medical records. We utilized a total of 389 documents from this i2b2 dataset, including 35 documents of *current smoker*, 66 of *non-smoker*, 36 of *past smoker*, and 252 of *unknown*. We utilized the NLP algorithm in case study 1 for identifying *non-smoker* and the algorithm presented in Table 3 for *current smoker* and *past smoker*.

**Table 3:** Keywords of the NLP algorithm for the extraction of smoking status in the i2b2 2006 shared task

Current Smoker	(does|has|continues to) smoked?, uses tobacco, active smoker, (current|currently) (smoker|smoking), current smoker, tobacco use\W*(yes|still using|still smoking|smokes)
Past Smoker	(stop|stopped|quit|quitted|discontinued) (tobacco|smoking), (previous|prior|remote|distant|former|ex-|ex) (tobacco|smoker), stop(ped)? smoking, tobacco use\W*(smoked|quit), smoking\W*(used|former)

## Results

### Results of the clinical text classification paradigm

Table 4 shows the results of the proposed paradigm compared with the rule-based NLP algorithms in the three case studies. In the first two institutional case studies, CNN achieved the best performance amongst the tested machine learning methods, and outperformed the rule-based NLP algorithms for both tasks with statistical significance. The results imply that CNN is able to capture hidden patterns from the weakly labeled training data that are not included in the rule-based NLP algorithms. SVM is inferior to CNN for the Mayo Clinic smoking status classification, but comparable to CNN for the proximal femur (hip) fracture classification. The performance of RF is worse than CNN for both classification tasks. RF performs better than SVM for the Mayo Clinic smoking status classification while worse for the proximal femur (hip) fracture classification. MLPNN performs worse than CNN but is comparable to SVM and RF in both tasks. The results from both experiments show that the CNN is the best fit in the proposed paradigm and could outperform the rule-based NLP algorithms.

**Table 4:** Comparison results of the proposed clinical text classification paradigm

Mayo Clinic Smoking Status Classification			
	Precision	Recall	F1 Score
Rule-based NLP	0.91	0.91	0.91
SVM	0.80	0.79	0.80
RF	0.82	0.81	0.81
MLPNN	0.85	0.85	0.85
CNN	**0.93** ^*^	**0.92** ^*^	**0.92** ^*^
Proximal Femur (Hip) Fracture Classification			
	Precision	Recall	F1 Score
Rule-based NLP	0.93	0.92	0.93
SVM	0.95	0.95	0.95
RF	0.93	0.93	0.93
MLPNN	0.95	0.95	0.95
CNN	**0.97** ^*^	**0.97** ^*^	**0.97** ^*^
i2b2 2006 Smoking Status Classification			
	Precision	Recall	F1 Score
Rule-based NLP	**0.91**	**0.89**	**0.88**
SVM	0.86	0.84	0.84
RF	0.85	0.84	0.83
MLPNN	0.83	0.82	0.82
CNN	0.76^*^	0.81^*^	0.77^*^

The asterisk indicates that difference between CNN and other methods is statistically significant

Unlike the first two institutional case studies, we observe that the proposed paradigm using four machine learning methods is not comparable to the rule-based NLP algorithm for the i2b2 smoking status classification shared task. CNN is inferior to other conventional machine learning methods and SVM achieves the best performance amongst the machine learning methods. The reason might be two-fold. First, the size of i2b2 dataset is too small for machine learning models to learn rules, particularly for CNN which requires a large dataset, while the NLP algorithm is developed for the task with manual handcrafted rules based on developer’s knowledge and experience. Second, the dataset is imbalanced for machine learning methods to learn latent rules for each category. For example, only 9% of the data was in the categories of current smoker and past smoker.

### Impact of the word embedding features

The results of incorporating word embeddings or other features are listed in Table 5. The machine learning models using word embedding features perform better than those using tf-idf and topic modeling features with statistical significance. The reason might be that word embedding features could alleviate the feature sparsity issues compared to tf-idf features [62], and represent better semantics compared to topic modeling features [63]. The models using topic modeling features are better than those using tf-idf features since topic modeling features contain semantic information of words. Since topic modeling requires prior distributions that are always difficult to define for a given corpus [64], its performance is usually inferior to word embeddings. This experiment verifies the advantage of word embeddings used as features for machine learning models in the proposed paradigm.

**Table 5:** Comparison of using different numbers of documents in the fracture task

Mayo Clinic Smoking Status Classification			
	tf-idf	topic modeling	word embeddings
SVM	0.69	0.73	0.80*
RF	0.69	0.72	0.81*
Proximal Femur (Hip) Fracture Classification			
	tf-idf	topic modeling	word embeddings
SVM	0.85	0.91	0.95*
RF	0.77	0.92	0.93*

The asterisk indicates that difference between word embeddings and other features is statistically significant

### Extra patterns learned by CNN

In this section, we compare the rule-based NLP algorithm with the clinical text classification paradigm using CNN, and demonstrate that additional hidden patterns could be captured by CNN compared to the rule-based NLP algorithm.

In the Mayo Clinic smoking status classification task, the information extracted by CNN and that by the rule-based NLP algorithm is different for 7 out of 475 testing cases. Among these 7 cases, CNN correctly identified the smoking status for 5 cases (71.4%). Table 6 lists three typical cases where the results of CNN and the rule-based NLP algorithm are different. In Case 1, the rule-based NLP algorithm failed due to the misspelled word “tobaco” in the clinical note not being considered in the rules. CNN was able to address this issue since it used word embedding features that represent the misspelled word in the neighborhood of the correct form in the semantic space [51]. The rule-based NLP incorrectly extracted non-smoker information from Case 2 due to the pattern “no smoking”. However, the whole statement “no smoking after age XXX” indicates a past smoker. This semantic meaning could be captured by CNN. CNN failed in Case 3 where the rule-based NLP algorithm correctly captured the correct smoking status due to the rules inspired by human experience. Many physicians write clinical notes following certain structures, which result in semi-structured clinical note, such as “Tobacco current use: No never used any” in Case 3. Since we were aware of this based on experts’ experience, the rules in the NLP algorithm could handle it properly. However, machine learning methods might focus on the pattern “Tobacco current use” and thus extracted the smoking status incorrectly.

**Table 6:** Cases from the institutional classification tasks where the results of CNN and the rule-based NLP algorithm are different

Task	Case #	Text Snippets	Gold Standard	Rule-based NLP	CNN
Mayo Clinic Smoking Status Classification	1	…She is a taxi driver and she has never used tobaco products…	Non-smoker	X	Y
	2	…No smoking after age XXX…	Smoker	X	Y
	3	…Tobacco current use: No never used any...	Non-smoker	Y	X
Proximal Femur (Hip) Fracture Classification	1	…Indications: femur fx…Cannulated screw fixation of the right femoral neck…	Proximal Femur fracture	X	Y
	2	… Pin fixation across the proximal left femoral neck…	Proximal Femur fracture	X	Y
	3	Exam: Sp Cerv*2vw Flex/Ext only Indications: Fx Vertebra Cervical Closed…	Non-Proximal Femur fracture	X	Y
	4	Exam: R Major Jnt Asp and/or Inj Indications: R hip inj/marc/steroid; fx femur neck nos closed, pain hip...	Proximal Femur fracture	Y	X

We use Y to indicate the correct extraction result and X otherwise

The proximal femur (hip) fracture identified by CNN differs from that by the rule-based NLP algorithm for 6 out of 498 testing cases. Among these 6 cases, CNN correctly identified the hip fracture status for 5 cases (83.3%). Similar to the Mayo Clinic smoking status classification task, a few typical cases are listed in Table 6 for the hip fracture classification task. In Case 1, the rule-based NLP algorithm failed to extract fracture information since the NLP failed to match the pattern of the keyword “fx” and “right femoral neck”, which occurred across sentences. CNN has no such issue since it does not require a sentence detection algorithm. Case 2 is likely a fracture in proximal femur where a percutaneous pin has been placed. Similar to Case 1 in the Mayo Clinic smoking status classification task, the rule-based NLP algorithm failed in Case 2 due to the missing fracture keyword “fracture” in the report. Unlike the rule-based NLP algorithm that entirely relies on the rules, CNN could correctly extract the fracture information since the representations of these keywords in the embedding space are semantically similar to “fracture”. Case 3 is not describing a proximal femur fracture based on the context. However, the rule-based NLP algorithm matched the rules in the sentence “Fx…cervical” and ignored the context in the middle. In contrast, CNN could take the context into account for calculating the document representation using word embedding features and accurately determined this is not a proximal femur fracture. The rule-based NLP algorithm correctly extracted fracture information for Cases 4 whereas CNN failed. The reason might be that the proximal femur fracture signal in this case is too weak for CNN as the document only mentions the fracture in the indication.

For the i2b2 2006 smoking status classification shared task, CNN differs from the rule-based NLP algorithm on 21 out of 79 testing cases among which CNN only correctly identified 5 cases (23.8%). Most of these 5 cases are *non-smoker* cases where CNN could detect the negation while the NLP algorithm does not consider certain specific rules, such as classifying “tobacco history: none” as *non-smoker*. Among the 16 cases where the NLP algorithm correctly identified the smoking status, 8 cases were categorized to *unknown* by CNN. This may be due to a large portion of *unknown* cases existing in the unbalanced training dataset (around 65%). In addition, CNN does not perform well on identification of cases that are small in terms of training data size. For example, only 4 *past smoker* cases in the whole dataset contain “remote smoker” as keywords. Thus, CNN failed to classify patients with mentions of “remote smoker history” in their clinical documents into *past smokers* while the NLP algorithm could correctly extract it due to the rule “(previous|prior|remote|distant|former|ex-|ex) (tobacco|smoker)”.

Table 7 lists some keywords in our institutional classification tasks and the selected semantically similar words found by the deep representation method. First, we observe that the deep representation could capture similar words regardless of morphological change. For example, “cigar”, “cigarettes”, and “cigars” are similar words to “cigarette”, “fx” is similar to “fracture”. More interestingly, we could find misspelled words similar to the correct forms, such as “nicotene” to “nicotine”, “cervial” to “cervical”, and “intratrochanteric” and “introchanteric” to “intertrochanteric”. Second, the deep representation method could find semantically similar words. For example, “cigarette” is semantically similar to “tobacco”; “intramedullary”, “intermedullary”, “nailing”, and “pinning” are related to surgical fixation of the hip fracture; “transtrochanteric”, “pertrochanteric”, “basicervical”, and “intertroch” are similar to the keyword “intertrochanteric” in terms of a description of the location of the proximal femur. Keywords of either different morphologies or semantics may not be easily identified by humans when developing a rule-based NLP algorithm.

**Table 7:** Keywords for the institutional classification tasks and the corresponding semantically similar words found by the deep representation method

Task	Keyword	Selected semantically similar words
Mayo Clinic Smoking Status Classification	smoke	secondhand, thirdhand, pipes, nutcrackers, cigs
	tobacco	cigarettes, cigarette, cigar, cigars, tobaco
	cigarette	cigar, hookah, tobacco, cigarettes, cigars
	nicotine	nicotene, nicoderm, nictoine
Proximal Femur (Hip) Fracture Classification	fx	fracture, comminuted, pinning, displaced, fractures
	intertrochanteric	intramedullary, nailing, pinning, intratrochanteric, introchanteric, transtrochanteric, pertrochanteric, basicervical, intertroch
	greater trochanter	trochanters, troch, trochanteric

## Discussion

Application of machine learning methods to clinical text classification tasks is hampered by the need for extensive human efforts to create large labeled training data sets and to conduct feature engineering [65]. The proposed paradigm could alleviate this problem by leveraging weak supervision and deep representation. In the weak supervision, the rule-based NLP algorithm was developed to automatically assign weak labels for training data. In the deep representation, word embeddings learned by deep neural networks were utilized as features. We have demonstrated that our approach can achieve high performance on binary clinical text classification tasks. The proposed approach could reduce human efforts in terms of data annotation and feature engineering. Since there are publicly available NLP algorithms [21] and pre-trained word embeddings “(https://github.com/3Top/word2vec-api)”, the proposed paradigm may be an easier way for non-experts to use machine learning methods for clinical text classification in healthcare institutions.

In the recent literature, the majority of clinical applications using weak supervision as a deep learning method have focused on medical image data. Since weak supervision does not require manual annotation, it becomes scalable for annotating a large scale of local patches for a medical image classification task. For example, Yan et al. [66] used the pre-trained CNN to automatically label a large set of patches for a bodypart multiclass image classification problem. Jia et al. [67] developed a weakly supervised learning algorithm to learn to segment cancerous regions in histopathology images. Madooei et al. [68] used weak supervision in a binary image classification task to categorize blue-white structures in dermoscopy images. Similar to our results, these studies attain performance close to machine learning approaches with supervision. Regarding using weak supervision on text data, researchers focused applications on biomedical texts, such as biomedical word sense disambiguation [69] and biomedical named entity recognition [70]. Unlike our approach that leveraged NLP algorithms, these approaches utilized external knowledge base to automatically generate labels. For example, Sabbir et al. [69] used the MSH WSD dataset for weak supervision and Fries et al. [70] used biomedical resources like lexicons in the weak supervision. Similarly, these approaches achieved competitive performance compared to state-of-the-art systems trained on hand-labeled data. Our proposed approach attempts to apply weak supervision to the clinical text data and provides a clinical text classification paradigm.

Our study shows that deep neural networks are robust to massive label noise and that sufficiently large training data is important for effectively training deep neural networks, which is consistent with a recent study in the common machine learning domain [71]. While the experimental comparison has shown the advantage of deep neural networks over the conventional machine learning approaches, it may also overestimate the performance of deep neural networks. The reason is that the neural network methods rely on a large training dataset and have been developed for more complex tasks rather than binary document classification. There is also improvement space for CNN on our clinical text classification tasks by tuning parameters, initialization methods and loss functions. However, it requires manual engineering and it is not guaranteed that the optimal parameters are generalizable to a different task.

We have empirically shown that the additional rules deep neural networks learned are based on the semantically similar words identified by the deep word embedding representation. These semantically similar words could be leveraged to augment the rules of the NLP algorithm in the future work. The deep representation also found noisy words that were irrelevant to the specific clinical text classification task. We would thus like to study how to eliminate noisy words identified by deep word embedding representation in the future work. In addition, other deep representation methods, such as character embeddings [72], are also subject to a future study.

Evaluation of the portability of the proposed paradigm is also an interesting topic. Since clinical practice and workflow vary across institutions, the performance of NLP systems varies across institutions and sources of data [73]. An NLP system performing well in one institution might need to redesign rules according to the sublanguage characteristic in the institutional EHR system, which requires lots of efforts. However, machine learning models may not need extra modification when switching from one institution to another as they learn rules automatically, which may significantly reduce implementation time and expenses. Therefore, the portability of the proposed paradigm across different institutions is an important research topic and subject to a future study.

A few questions regarding the theory of the proposed paradigm remains open. For example, it is not clear how simple an NLP algorithm (i.e., how many rules) is sufficient for machine learning methods and what accuracy an NLP algorithm should have (i.e., how small should *ϵ* be) to generate useful weak labels.

### Limitations

**Fig. 4:**
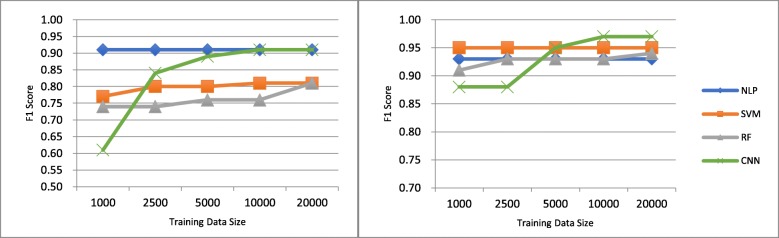
Comparison of using different sizes of training dataset for Mayo Clinic Smoking Status Classification (left figure) and Proximal Femur (Hip) Fracture Classification (right figure). Note: The vertical axis represents the size of training data. The vertical axis represents the F1 score

The first limitation of this study is that CNN is sensitive to the data size. One question we are interested is: do we really need the entire dataset of 31,861 clinical notes for training in the Mayo Clinic smoking status classification task or that of 22,471 radiology reports for training in the proximal femur (hip) fracture classification task? In order to answer this question, we tested the proposed paradigm by using different sizes of training data, namely 1000, 2500, 5000, 10,000, and 20,000. Note that these training data were randomly sampled from the entire dataset. Figure 4 depicts the F1 score curves of machine learning methods and the rule-based NLP algorithms for the Mayo Clinic smoking status classification and proximal femur (hip) fracture classification tasks. When the data size is 1000, SVM and RF outperform CNN in both tasks. When the data size increases to 5000, the performance of CNN increases dramatically and becomes better than SVM and RF in both tasks. As the data size becomes 10,000, CNN does not have much performance gain compared to the data size of 5000, but it outperforms the rule-based NLP algorithm for both tasks. When the data size is 20,000, the performance of CNN is the same as that when the data size is 10,000. The performance curves of CNN clearly show that this deep learning method is more sensitive to the data size, while the rule-based NLP algorithms and the conventional machine learning methods are more resistant to the data size. This result also explains why CNN underperforms the conventional machine learning methods for the i2b2 2006 smoking status classification shared task. We can also see that 5000 documents (about 20%) of training data might be sufficient for CNN to learn most extraction patterns for a single concept. However, how much data is sufficient for training CNN is still an open question and needs further research.

The second limitation of this study is that the proposed paradigm was shown effective for simple binary clinical text classification tasks that aim to extract single concepts but less effective for complex multiclass clinical text classification tasks that aim to extract multiple concepts, which is shown by the case study of i2b2 2006 smoking status classification shared task. This limitation may be due to the small size of training data and imbalanced i2b2 dataset. Therefore, we will create a large multiclass clinical text dataset to evaluate the proposed paradigm for extracting multiple concepts in the future.

Though deep learning models have achieved the state-of-the-art performance on several NLP tasks, the third limitation of this study is that deep learning models inherently lack interpretability compared to the rule-based algorithms. In this study, we have shown this drawback in the error analysis. The rule-based algorithms are easy to interpret and rules can be added or modified easily. However, the rule-based algorithms require extensive human efforts to develop as well as expert knowledge and experience. As a matter of fact, researchers in the deep learning domain have conducted preliminary work to interpret deep models and deep representation features. For example, Erhan et al. [74] interpreted deep networks by visualizing the hierarchical representations learned by deep networks. Che et al. [75] introduced a powerful knowledge-distillation approach to learn interpretable deep learning models. Therefore, we will study the interpretability of the proposed paradigm and compare it with the rule-based algorithms for clinical text classification tasks in the future.

## Conclusions

In this paper, we proposed a clinical text classification paradigm using weak supervision and deep representation. In this paradigm, we first developed a rule-based NLP algorithm to automatically generate labels for the training data, and then used the pre-trained word embeddings as deep representation features to eliminate the need for task-specific feature engineering for training machine learning models. Although the training data was weakly labeled, we theoretically showed that machine learning models trained from these weak labels could achieve similar training performance to that trained from true labels. We validated the effectiveness of the proposed paradigm using two institutional case studies at Mayo Clinic: smoking status classification and proximal femur (hip) fracture classification, and one case study using a public dataset: the i2b2 2006 smoking status classification shared task. We tested four prevalent machine learning models, i.e., SVM, RF, MLPNN and CNN. The results from both institutional experiments show that the CNN is the best fit in the proposed paradigm that could outperform the rule-based NLP algorithms. We showed that word embeddings significantly outperformed tf-idf and topic modeling features in the paradigm, and that CNN could capture additional patterns from the weak supervision compared to the rule-based NLP algorithms. We also overserved two drawbacks of the proposed paradigm. One is that CNN is more sensitive to the size of training data than the rule-based NLP algorithm and the conventional machine learning methods. The other drawback is that the proposed paradigm might not be competitively effective for complex multiclass clinical text classification tasks, as shown by the case study of i2b2 2006 smoking status classification shared task.

## Abbreviations

CNN: Convolutional Neural Networks
EHR: Electronic Health Record
HITECH: Health Information Technology for Economic and Clinical Health
LDA: Latent Dirichlet Allocation
MLPNN: Multilayer Perceptron Neural Networks
NLP: Natural language processing
RF: Random Forrest
SVM: Support Vector Machine
